# 3-D Flow Reconstruction Using Divergence-Free Interpolation of Multiple 2-D Contrast-Enhanced Ultrasound Particle Imaging Velocimetry Measurements

**DOI:** 10.1016/j.ultrasmedbio.2018.10.031

**Published:** 2019-03

**Authors:** Xinhuan Zhou, Virginie Papadopoulou, Chee Hau Leow, Peter Vincent, Meng-Xing Tang

**Affiliations:** ⁎ Department of Bioengineering, Imperial College London, London, United Kingdom; † Joint Department of Biomedical Engineering, University of North Carolina, Chapel Hill, and North Carolina State University, Raleigh, North Carolina, USA; ‡ Department of Aeronautics, Imperial College London, London, United Kingdom

**Keywords:** 3-D flow reconstruction, Divergence-free interpolation, Ultrafast Contrast-enhanced ultrasound imaging velocimetry, PIV

## Abstract

Quantification of 3-D intravascular flow is valuable for studying arterial wall diseases but currently there is a lack of effective clinical tools for this purpose. Divergence-free interpolation (DFI) using radial basis function (RBF) is an emerging approach for full-field flow reconstruction using experimental sparse flow field samples. Previous DFI reconstructs full-field flow from scattered 3-D velocity input obtained using phase-contrast magnetic resonance imaging with low temporal resolution. In this study, a new DFI algorithm is proposed to reconstruct full-field flow from scattered 2-D in-plane velocity vectors obtained using ultrafast contrast-enhanced ultrasound (>1000 fps) and particle imaging velocimetry. The full 3-D flow field is represented by a sum of weighted divergence-free RBFs in space. Because the acquired velocity vectors are only in 2-D and hence the problem is ill-conditioned, a regularized solution of the RBF weighting is achieved through singular value decomposition (SVD) and the L-curve method. The effectiveness of the algorithm is determined *via* numerical experiments for Poiseuille flow and helical flow with added noise, and it is found that an accuracy as high as 95.6% can be achieved for Poiseuille flow (with 5% input noise). Experimental feasibility is also determined by reconstructing full-field 3-D flow from experimental 2-D ultrasound image velocimetry measurements in a carotid bifurcation phantom. The method is typically faster for a range of problems compared with computational fluid dynamics, and has been found to be effective for the three flow cases.

## Introduction

Atherosclerosis, caused by the buildup of atheromas in the lining of artery walls and narrowing arteries, can affect any artery in the body, causing diseases such as angina, stroke, heart attack and peripheral artery disease. Development of atherosclerosis is associated with bends and bifurcations in vessel geometry, and it has been hypothesized that flow patterns related to differences in geometry and wall shear stress are involved in the selective localization of atherosclerosis (Glagov et al., 1988, Ku et al., 1985, Zarins et al., 1983). Studying physiologic flow patterns in complex geometries where atherosclerosis is more likely to appear may elucidate the mechanisms that lead to its development.

Computational fluid dynamics (CFD) is a standard physiologic flow simulation tool. High-fidelity CFD requires (i) accurate flow domain geometry, which poses a challenge for lumen surface reconstruction from noisy and scattered imaging input, because in many cases imaging input includes missing boundary data with holes resulting from the accessibility limitation of imaging scanners; (ii) reliable 3-D boundary/initial conditions and fluid properties (accounting for the non-Newtonian rheology of blood) (Johnston et al., 2004, Yilmaz and Gundogdu, 2008). In addition, the computational cost of adequately resolving the physics of complex flows by traditional CFD is high, while it is challenging to predict complex flows using low-fidelity models with confidence. Even with the recent progress in parallel computing, fast CFD has only been possible under restricted conditions (Geveler et al., 2011, Liu et al., 2004, Wu et al., 2004).

Ultrasound imaging is highly accessible and affordable compared with magnetic resonance imaging and has been used in the study of atherosclerosis to (i) quantify arterial wall thickness as a measure of atherosclerosis risk (Chambless et al., 1997, Gussenhoven et al., 1989, Pignoli et al., 1986); (ii) acquire flow domain geometry and conduct CFD (Augst et al., 2003, Lee et al., 2004, Zhao et al., 2000); and (iii) measure 2-D flow velocity by speckle tracking (Leow and Tang, 2018, Leow et al., 2015, Nam et al., 2012, Niu et al., 2010) or velocity Doppler (Fox, 1978, Overbeck et al., 1992, Scabia et al., 2000) and correlate flow field with atherosclerosis risk. Multibeam Doppler estimation methods measure the Doppler frequency from different beam-flow angles and solve the individual flow vector components (Fox, 1978, Overbeck et al., 1992). Speckle tracking using microbubble contrast agents (also known as echo particle image velocimetry [echo-PIV] or ultrasound image velocimetry [UIV]) is another method that can quantify 2-D flow and perfusion within blood vessels and heart ventricles (Kim et al., 2004, Poelma et al., 2011, Toulemonde et al., 2017) by combining microbubble contrast agents and ultrasound. With the advancement of high-frame-rate plane wave ultrasound imaging (typical frame rate: >1000 fps), the temporally changing features of fast and pulsatile flow can be fully resolved. Although ultrasound imaging is still primarily in two dimensions, with contrast-enhanced ultrafast UIV it is possible to acquire the 3-D geometries of vessels and the ventricles by stacking 2-D images slice by slice, as well as two-dimensional in plane velocity in optically opaque geometries. A detailed experimental setup and post-processing algorithm of plane wave ultrafast UIV were recently introduced by Leow and co-workers (Leow and Tang, 2018, Leow et al., 2015). However, like most velocimetry imaging techniques, plane wave ultrafast UIV obtains 2-D in-plane velocity fields at discrete imaging planes and the out-of-plane velocity component is lost. So it is of much benefit and interest to build full 3-D flow fields from such scattered 2-D velocity data with a reliable and computationally cheap method.

Divergence-free interpolation (DFI) using radial basis function (RBF) is a mesh-free flow reconstruction algorithm of low computational cost, and we expect it to have high accuracy as it reconstructs the flow field from experimental data. As an alternative to CFD, it combines 3-D vectorial data with a mass conservation law, and it does not require fluid properties such as viscosity, or even the full geometry, to be known. It is therefore more reliable than CFD in cases when an accurate boundary/initial condition and geometry are unavailable (Lowitzsch, 2005a, Lowitzsch, 2005b, Wendland, 2009). DFI was first proposed by Narcowich and Ward (1994) and was then proven accurate in obtaining full-field flow velocity by Lowitzsch, 2004, Lowitzsch, 2005a. Chan and Liebling (2015) recently proposed a reconstruction method from multiple directional PIV measurements in optical microscopy. Skrinjar et al. (2009) and Sundareswaran et al. (2012) reported on the feasibility of DFI using 3-D velocity input from phase-contrast magnetic resonance imaging. However, previous DFI techniques depended on 3-D sparse velocity input for flow reconstruction and cannot work on 2-D velocity measurements by ultrasound.

In this study, we proposed a new 3-D flow reconstruction method using 2-D in-plane projected vectorial data, taking advantage of ultrafast plane wave UIV at a high frame rate (>1000 fps) and divergence-free RBF. As far as we know this is the first study able to reconstruct the full flow field from 2-D flow measurements and to reconstruct 3-D flow using ultrafast UIV.

The experimental method and basic theories of ultrasound-augmented DFI (UADFI) are introduced first, followed by numerical simulation on a straight vessel flow and a helical flow and, finally, in a carotid bifurcation phantom. Appendix A introduces four different RBF kernels, and Appendix B compares three algorithms to select the optimal regularization parameter for the UADFI system. Grid convergence of the helical flow using STAR-CCM+ (Version 11.06, Siemens, Berlin, Germany) is described in Appendix C.

## Methods

### Data acquisition setup (simulation and experiments)

**Fig. 1:**
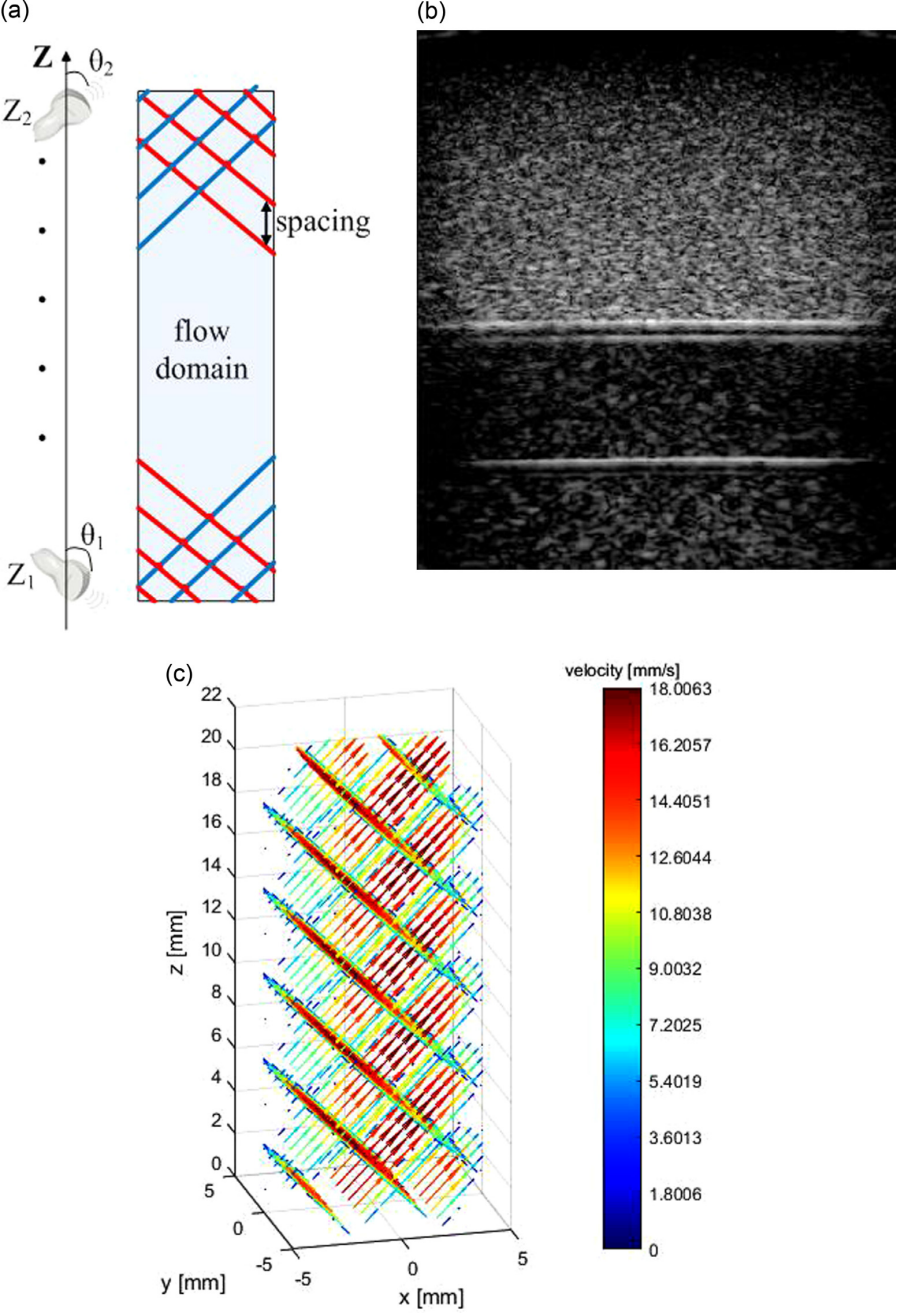
(a) Schematic of straight cylindrical vessel (top view) along the probe motion direction, with *blue* and *red* imaging planes demarcating the two independent scanning directions. (b) One B-mode image acquired in a straight vessel. (c) Illustration of 2-D velocity acquired in a straight vessel by ultrasound imaging velocimetry.

In UADFI, multi-angle 2-D velocimetry measurements are acquired (or simulated). In this study, two distinct acquisition angles were required because these provide the projections of the real 3-D flow field onto two plane directions, which contain independent flow information to allow for full-field reconstruction. The angles between the imaging plane (probe plane) and probe motion direction are denoted as θ_1_ and θ_2_ for two imaging directions, respectively. In practice, ultrasound scanning is achieved as follows: after scanning at discrete planes separated by spacing along the *Z*-axis (defined along the direction of the probe motion [Fig. 1a]) for the first angle (θ_1_) from *Z*_1_ to *Z*_2_, the imaging probe was rotated to θ_2_, and the same flow domain was scanned along the –Z direction until the probe reached *Z*_1_ (Fig. 1a). The motion of the probe is controlled by a programmed stage controller, and its step size is 5 mm (spacing = 5 mm). Acquisition angles (θ_1_ = 45°, θ_1_ = 135°) and spacing are illustrated in Figure 1. Two-dimensional velocity vectors were then registered according to acquisition parameters and segmented by a level set method (Lee et al. 2016), as illustrated in Figure 1b.

### Reconstruction algorithm

Blood flow is usually assumed incompressible (Oshima et al., 2001, Tu and Deville, 1996), and the divergence of its velocity is zero. DFI with 3-D velocity input was introduced in some previous work (Lowitzsch, 2004, Lowitzsch, 2005b; Narcowich and Ward, 1994, Wendland, 2009), and we briefly introduce the previous method below in eqns (1)–(4) and our new algorithm in eqns (5) to (11).

#### Existing algorithm: Divergence-free interpolation with 3-D velocity input

RBF is a family of scalar valued approximation functions used for interpolation. The value of RBF at a point $\vec{x}$ depends on its distance from the interpolation centre $\vec{c}$. The divergence-free matrix-valued RBF, $\emptyset (\vec{r})$, is defined by

(1) $$\emptyset \left(\vec{r}\right)=\left(-\Delta I+\nabla \nabla^{T}\right)\varphi \left(\vec{r}\right)$$

where $\varphi (\vec{r})$ is the scalar valued RBF (see examples of such RBFs in Appendix A) and $\vec{r}=\vec{c}-\vec{x}$. *I* is the identity matrix. If we choose the 3-D Gaussian function as the scalar valued RBF, eqn 1 can be expanded as

(2) $$\emptyset \left({\vec{x}}_{j},{\vec{c}}_{i}\right)=\left\{\left(6ɛ-4{ɛ}^{2}{∥\vec{r}∥}^{2}\right)I+4{ɛ}^{2}\vec{r}{\vec{r}}^{T}\right\}\text{exp}\left(-ɛ{\vec{r}}^{2}\right)$$

where ɛ is the reciprocal of the variance of the Gaussian function, here referred to as the shape parameter of Gaussian (see Appendix A), and $\emptyset ({\vec{x}}_{j},{\vec{c}}_{i})$ is a 3 × 3 symmetric matrix. ${\vec{x}}_{j}$ is the coordinate of the *j*th velocity vector, and ${\vec{c}}_{i}$ is the coordinate of the *i*th interpolation center point. For simplification, the point cloud of center points coincides with that of measurement data points. Then the velocity interpolation scheme has the form

(3) $$\vec{v}\left({\vec{x}}_{j}\right)=\sum_{i\;=\;1}^{m}\emptyset \left({\vec{x}}_{j},{\vec{c}}_{i}\right)\lambda_{i}$$

where *m* is the number of interpolation centers, and $\lambda_{i}={\left[\lambda_{i1},\lambda_{i2},\lambda_{i3}\right]}^{T}$ is a vector of weight coefficient for the RBFs. Each column of $\emptyset ({\vec{x}}_{j},{\vec{c}}_{i})$ is divergence free *via* eqn (1), and the interpolated velocity $\vec{v}\left({\vec{x}}_{j}\right)$ is a linear combination of divergence-free columns (see eqn [3]) and is thus divergence free (Narcowich and Ward 1994). We then rewrite eqn (3) in a compact matrix form:

(4) $$\underset{\vec{v}}{\underset{︸}{\left[\begin{array}{c} \vec{v}\left({\vec{x}}_{1}\right)\\ \vdots \\ \vec{v}\left({\vec{x}}_{m}\right) \end{array}\right]}}=\underset{G}{\underset{︸}{\left[\begin{array}{ccc} \emptyset \left({\vec{x}}_{1},{\vec{c}}_{1}\right) & \cdots & \emptyset \left({\vec{x}}_{1},{\vec{c}}_{m}\right)\\ \vdots & \ddots & \vdots \\ \emptyset \left({\vec{x}}_{m},{\vec{c}}_{1}\right) & \cdots & \emptyset \left({\vec{x}}_{m},{\vec{c}}_{m}\right) \end{array}\right]}}*\underset{\lambda }{\underset{︸}{\left[\begin{array}{c} \lambda_{1}\\ \vdots \\ \lambda_{m} \end{array}\right]}}$$

Once eqn (4) is solved and weight coefficients *λ* are obtained, 3-D velocity vectors at any spatial location within the flow domain can be computed with eqn (3). All previous DFIs are based on matrix inversion of eqn (4) using 3-D velocity measurement input $\vec{v}$, not suitable for UIV where only 2-D in-plane velocities are available.

#### New algorithm: Divergence-free interpolation with 2-D velocity input

Based on the previous work on DFI, we derived the new algorithm of UADFI based on 2-D UIV velocity input, discussed in the following four subsections.

##### Projection matrix

Assume a velocity vector $\vec{A}={\left(A_{x},A_{y},A_{z}\right)}^{T}$, a plane whose orientation is defined by its normal vector $\vec{B}={\left(B_{x},B_{y},B_{z}\right)}^{T}$. The projection of $\vec{A}$ on the plane, denoted by $\vec{A}∥\vec{B}$, is

(5) $$\vec{A}∥\vec{B}=\underset{R_{1}\left(or\;R_{2}\right)}{\underset{︸}{\left[\begin{array}{ccc} B_{y}^{2}+B_{z}^{2} & -B_{x}*B_{y} & -B_{x}*B_{z}\\ -B_{x}*B_{y} & B_{x}^{2}+B_{z}^{2} & -B_{y}*B_{z}\\ -B_{x}*B_{z} & -B_{y}*B_{z} & B_{x}^{2}+B_{y}^{2} \end{array}\right]}}\left[\begin{array}{c} A_{x}\\ A_{y}\\ A_{z} \end{array}\right]$$

where the 3 × 3 square matrix on the right-hand side is the projection matrix for the vector $\vec{A}$, which depends only on the direction of $\vec{B}$. This means only the angle between the imaging plane and probe motion direction is needed to construct the projection matrix. In this study two different imaging angles were used, and thus two 3 × 3 projection matrices *R*_1_ and *R*_2_ are constructed for each angle. Assume *m*_1_ and *m*_2_ are numbers of vectorial data points from imaging angles 1 and 2 ($m_{1}+m_{2}=m$). The projection matrix *R* for the UADFI system is a 3*m* × 3*m* block diagonal matrix, with *m*_1_ diagonal blocks of *R*_1_ and *m*_2_ diagonal blocks of *R*_2_:

(6) $$R=\left[\begin{array}{cc} \begin{array}{ccc} R_{1} & & \\ & \ddots & \\ & & R_{1} \end{array} & \\ & \begin{array}{ccc} R_{2} & & \\ & \ddots & \\ & & R_{2} \end{array} \end{array}\right]$$

##### Ultrasound-augmented divergence-free interpolation

By multiplying both sides of eqn (4) by projection matrix *R*, we have

(7) Aλ=b

where A=R*G and $b=R*\vec{v}$. *b* is the planar projection of true 3-D flow vector on two imaging planes, that is, velocity from UIV measurement. To reconstruct full-field 3-D flow by eqn (3), we aimed at finding a stable solution of *λ* for eqn (7).

##### Singularity and regularization for UADFI

As *R* is singular and rank(*R*) ≤ 3*m, A* is also singular because of the rank inequality:

(8) rank(R*G)≤min(rank(R),rank(G))≤3m

The condition number of the singular matrix *A* is infinite. As a result, small perturbations in *b* could result in arbitrarily large perturbations in *λ* when conducting matrix inversion of eqn (7). In real medical imaging applications, errors include measurement error, UIV post-processing and round-off errors, and are inevitable. In this study, to solve the ill-posed system of eqn (7) and obtain a stable and accurate solution of *λ*, regularization should be used, and below we introduce a stable truncated singular value decomposition (TSVD) solution to the noise-contaminated singular system in eqn (7).

##### TSVD pseudo-inverse

Velocity vector *b* is contaminated by experimental error, and eqn (7) becomes an error minimization problem

(9) $${}_{\;\lambda }^{\text{argmin}}\left\{{∥b-A\lambda ∥}^{2}+\tau \lambda^{2}\right\}$$

where *τλ*^2^ is the regularization term. To find the solution to eqn (9), we conduct SVD for *A*:

(10) $$A=U\Sigma V^{T}=\sum_{i\;=\;1}^{k}u_{i}\sigma_{i}v_{i}^{T}$$

Here, *U* and *V* are left singular vectors and right singular vectors, respectively; *σ_i_* is the *i*th singular value of *A; k* is the regularization parameter; and Σ is a diagonal matrix composed of singular values in descending order. Then a regularized solution of eqn (9) is

(11) $$\lambda_{\text{reg}}=\sum_{i\;=\;1}^{k}\frac{u_{i}^{T}bv_{i}}{\sigma_{i}}$$

*k* (*k* < rank(*A*)) acts as a measure of the extent to which the noise-contaminated UIV input should be trusted. Small *k* means the UIV input is less trusted and results in a large regularization error, whereas large *k* causes the system to be sensitive to noise and suffer large perturbation error. An optimal *k* to balance the regularization error and perturbation error is determined by the L-curve (Hansen and O'Leary 1993) method, and different methods to optimize the regularization parameter are compared in Appendix B.

After solving the regularized weight coefficient *λ*_reg_ with (11), (3) 3-D full-field flow is reconstructed with eqn (3). The UIV experimental setup is introduced below.

### Experimental measurement

Decafluorobutane microbubbles were diluted in water as contrast agent to a concentration of 2  × 10^5^ microbubbles/mL, and an L12-3v linear array probe connected to a Vantage 128 platform (Verasonics, Redmond, WA, USA) was used for UIV acquisition. A high-frame-rate plane wave pulse inverse scheme, with five compounding imaging angles between –9° and 9°, was transmitted, and the frame rate is 1000 fps. Then received RF data are beamformed by a delay and sum method to produce B-mode images. An autocorrelation technique developed by Leow et al. (2015) was used for 2-D in-plane flow velocity quantification. The 2-D experimental setup and acquisition procedures are described in Leow et al. (2015). In this study, the ultrafast UIV system was used to track steady flow in a carotid bifurcation tissue-mimicking phantom; it takes a matter of microseconds to acquire one image and several seconds for UIV post-processing using GPU, that is, in-plane velocimetry calculation.

### Three numerical/experimental cases

In this study, UADFI was tested on three steady flow cases below. Input for cases 1 and 2 was obtained by first extracting 3-D velocity vectors on two sets of imaging planes from an analytical solution or CFD, and then calculating their in-plane components.

• Case 1 (simulated case): Fully developed Poiseuille flow in a straight vessel with a diameter of 10 mm and length of 10 mm. The volumetric flow rate *Q* is 1 mL/s. In-plane UIV resolution is 1 mm.
• Case 2 (simulated case): Helical flow where the helix radius is 5 mm and total length is 35 mm. Inlet flow velocity is uniform at 10 mm/s (mean flow rate = 10 mm/s). In-plane UIV resolution is 1 mm.
• Case 3 (experimental case): Steady flow in a carotid bifurcation phantom with experimental 2-D UIV measurements. The inlet flow rate is 0.4 mL/s, and in-plane UIV resolution is 1 mm.

**Fig. 2:**
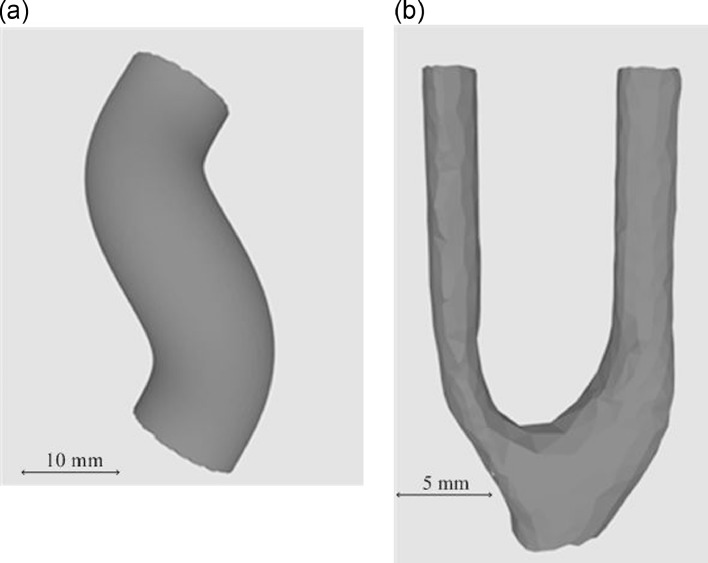
Geometries of cases 2 and 3: (a) helix; (b) carotid bifurcation (surface geometry from ultrasound scan).

**Fig. 3:**
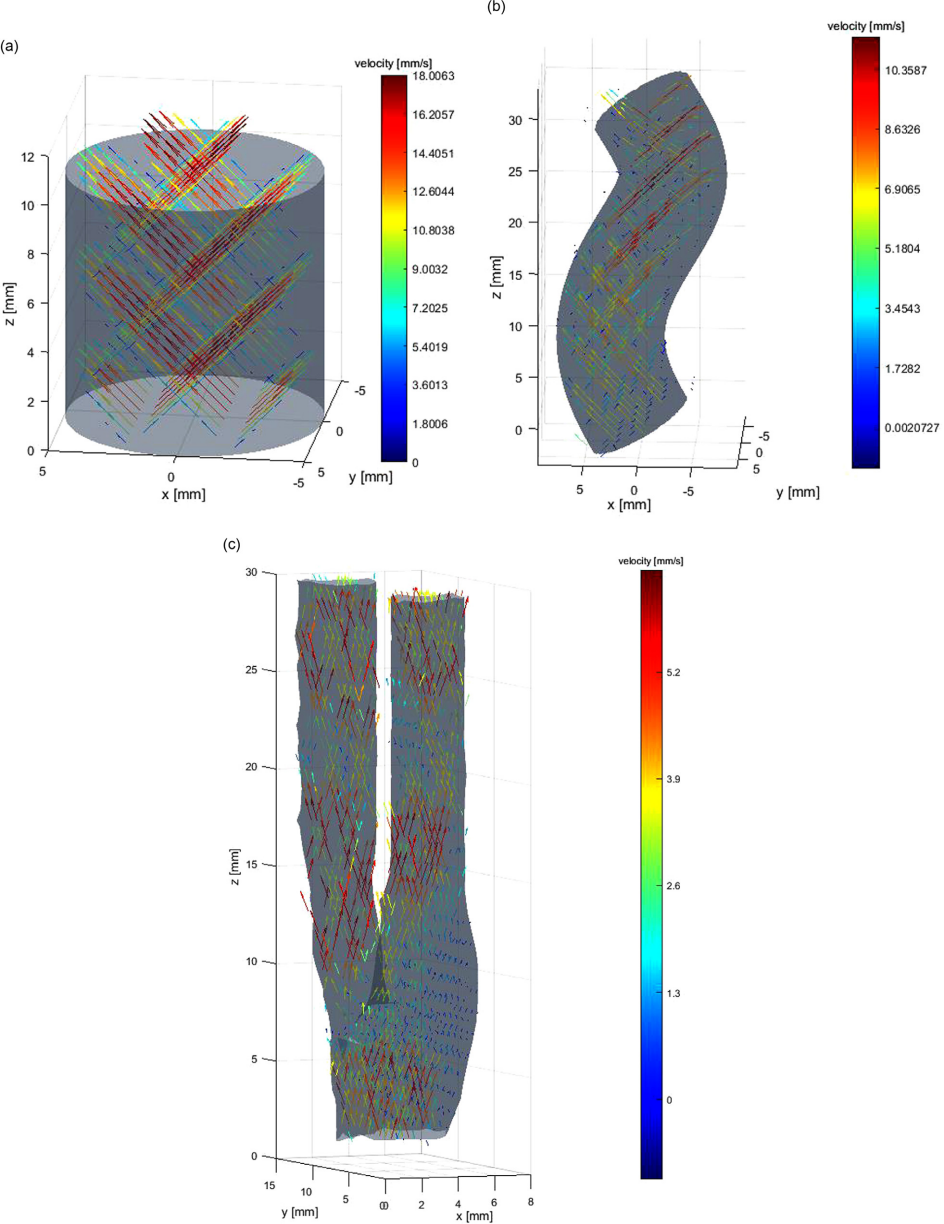
Input 2-D velocity for reconstruction of the three cases: (a) straight tube; (b) helix; (c) carotid bifurcation.

The geometries of cases 2 and 3 are illustrated in Figure 2, and the projected 2-D velocity after image registration is illustrated in Figure 3 (plane spacing = 5 mm in Fig. 3). The Reynolds numbers for the three cases are smaller than 1000. The helical flow CFD simulation is conducted in STAR-CCM+, and a steady, incompressible laminar flow model is used to simulate the flow. Grid independence is discussed in Appendix C. Errors of cases 1 and 2 were analyzed by comparing reconstructed flow with the Poiseuille equation and CFD, respectively. A 5% random artificial Gaussian error is then added to the sampled 2-D velocity of the first two cases to test the stability of the algorithm. In cases 1 and 2, different RBF kernels are also compared, and the influence of spacing on accuracy is investigated. All three cases in this study were conducted at double precision in MATLAB R2016 b (The MathWorks, Natick, MA, USA). All simulations were performed with an Intel Core i7 3.4-GHz HP EliteDesk 800 G1 Tower workstation with 32 GB RAM memory and NVIDIA GeForce GTX 1050 Ti GPU.

### Error analysis

The absolute error err is defined by

(12) $$\text{err}=\sqrt{\frac{\sum_{i\;=\;1}^{m}\left\{{\left(ut_{i}-u_{i}\right)}^{2}+{\left(vt_{i}-v_{i}\right)}^{2}+{\left(wt_{i}-w_{i}\right)}^{2}\right\}}{3m}}$$

where *ut_i_, vt_i_* and *wt_i_* are the three velocity components from ground truth, that is, CFD, and *u_i_, v_i_* and *w_i_* are velocities reconstructed by UADFI.

## Results

### Poiseuille flow case

**Fig. 4:**
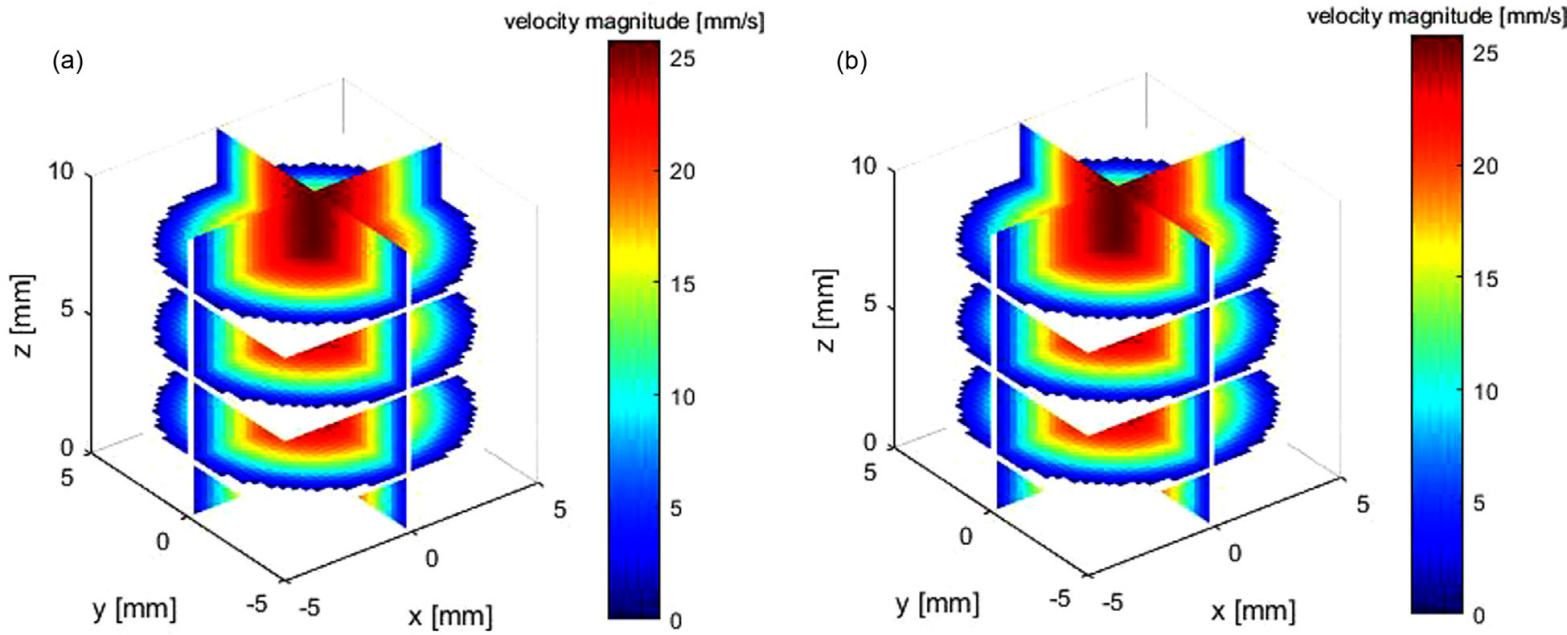
Poiseuille flow: (a) ground truth; (b) reconstructed flow field by Gaussian radial basis function.

**Fig. 5:**
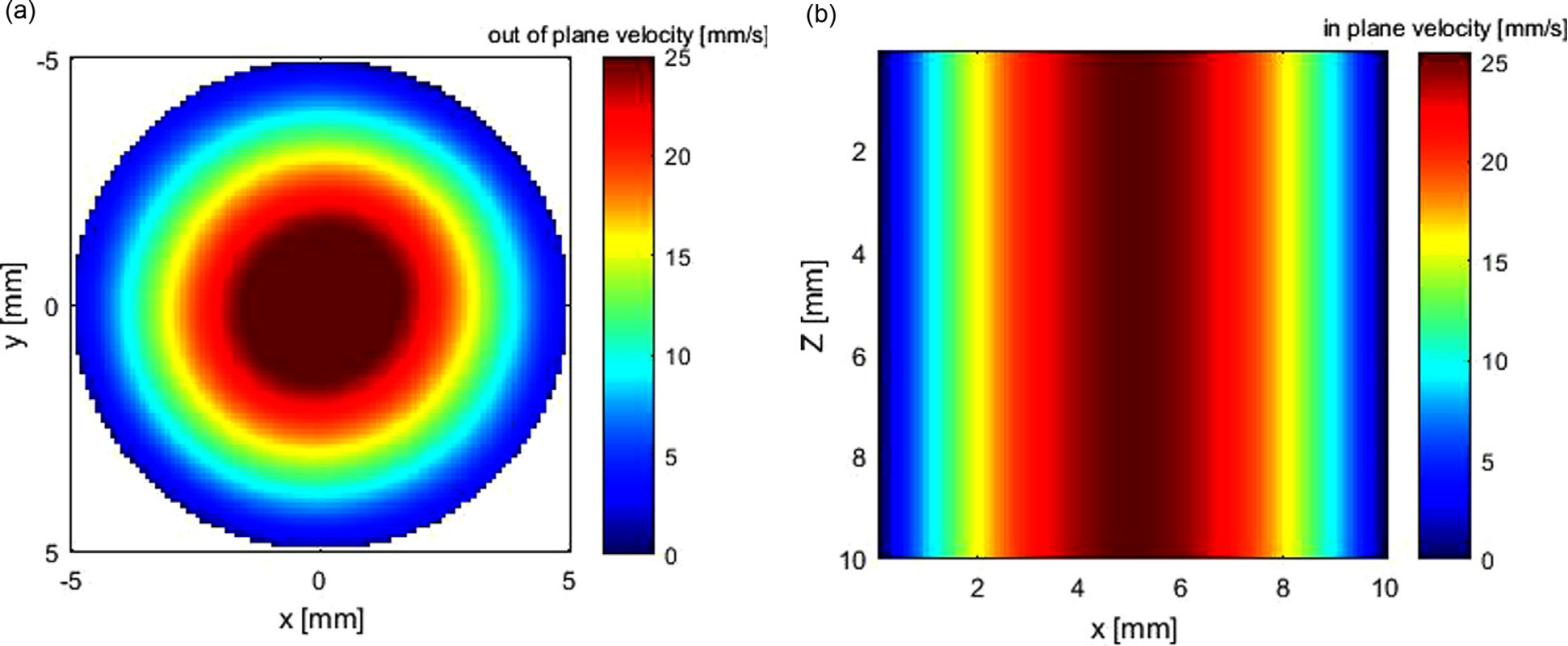
Poiseuille flow: (a) out-of-plane velocity at cross plane; (b) in-plane velocity at sagittal plane.

**Fig. 6:**
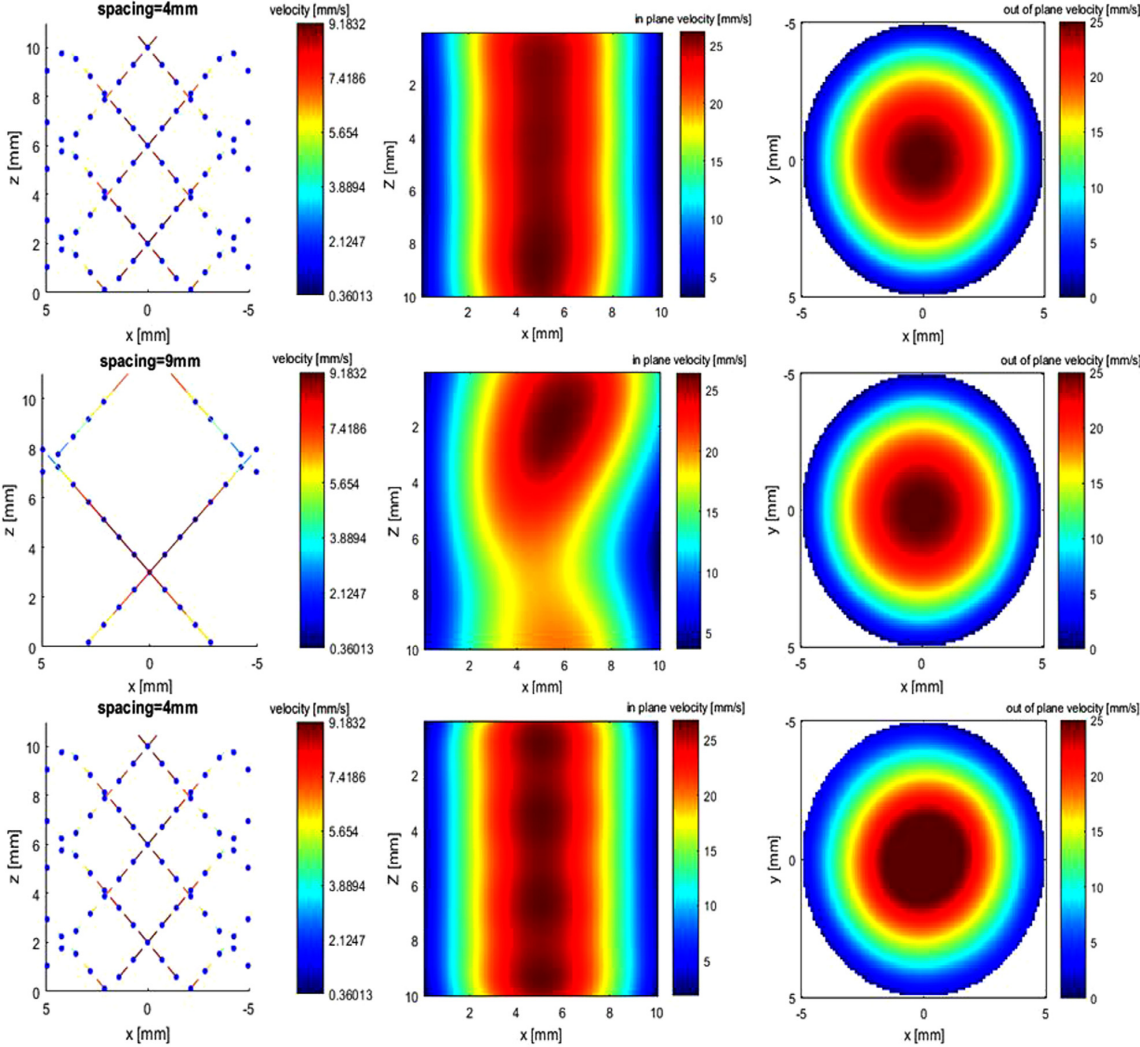
Poiseuille flow. The first two rows are interpolated flow using Gaussian kernel at spacings of 4 and 9 mm, respectively. The last row is using thin-plate spine kernel at a spacing of 4 mm. The three columns are sampled velocity, in-plane velocity at the sagittal plane and out-of-plane velocity at the cross plane.

Reconstruction results are illustrated in Fig. 4, Fig. 5, Fig. 6. As in practical applications, in-plane resolution is kept constant, and the influence of different RBF kernels and spacing between ultrasound acquisition planes on reconstruction accuracy is studied below.

#### Reconstruction accuracy using different kernels

Four different kernels are used (spacing = 2 mm) and it is outlined in Table 1 that Gaussian, inverse multiquadric (IM) and multiquadric (MU) have higher reconstruction accuracy than the thin-plate spine (TPS) kernel.

**Table 1:** Absolute error of four kernels for Poiseuille flow reconstruction using noise-free input

Kernel	Error (mm/s)
Gaussian	0.0399
IM (inverse multiquadric)	3.0652*e*-04
Multiquadric	5.8330*e*-04
TPS (thin-plate spline)	0.6358

##### Reconstruction accuracy using different spacing

**Fig. 7:**
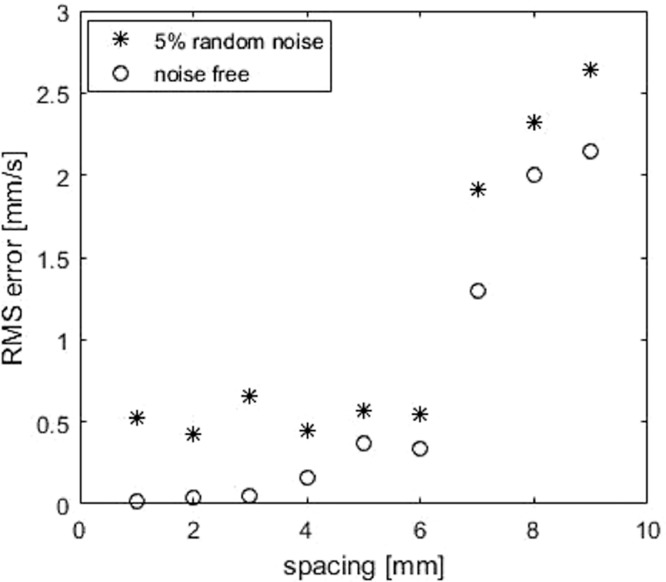
RMS error of Poiseuille flow at different spacings by Gaussian kernel, with or without 5% random noise. RMS = root mean square.

To reduce the duration of ultrasound acquisition and UIV post-processing, it is of great interest to increase the spacing between acquisition planes and evaluate the accuracy when spacing is large. The acquisition time constraint is especially important in the case of contrast-enhanced ultrasound imaging so that acquisitions in all necessary planes for reconstruction have to be within the current U.S. Food and Drug Administration limits of contrast agent administration in humans. Spacing is varied from 1 to 9 mm. It is shown in Figure 7 that increasing spacing reduces the reconstruction accuracy.

Ultrasound-augmented DFI is ill-conditioned, and to evaluate its numerical stability, 5% random Gaussian noise is added to the 2-D velocity input; that is, ǁ*b* – $\hat{b}$ǁ_2_/ǁ $\hat{b}$ǁ_2_ = 5%. The error of reconstructed flow, shown in Figure 7, indicates that the algorithm is robust with measurement noise, and err increases when spacing is larger. The relative error with 5% noise input increases from 4.4% to 20% (mean flow rate = 12.73 mm/s) when spacing increases from 1 to 9 mm.

### Helical flow case

#### Reconstruction accuracy using different kernels

**Fig. 8:**
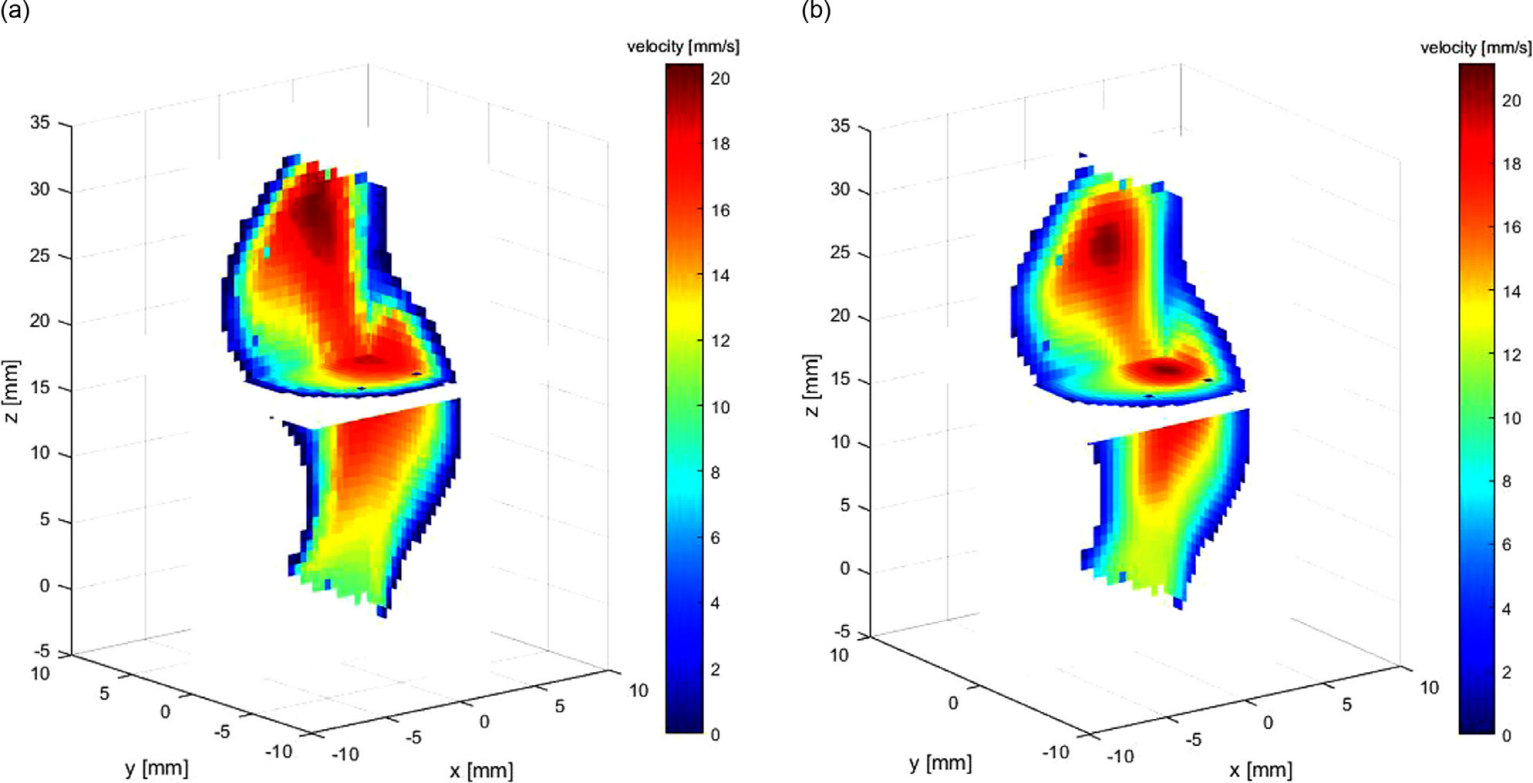
(a) Three-dimensional velocity of helical flow by computational fluid dynamics. (b) Reconstructed 3-D flow by Gaussian radial basis function.

**Fig. 9:**
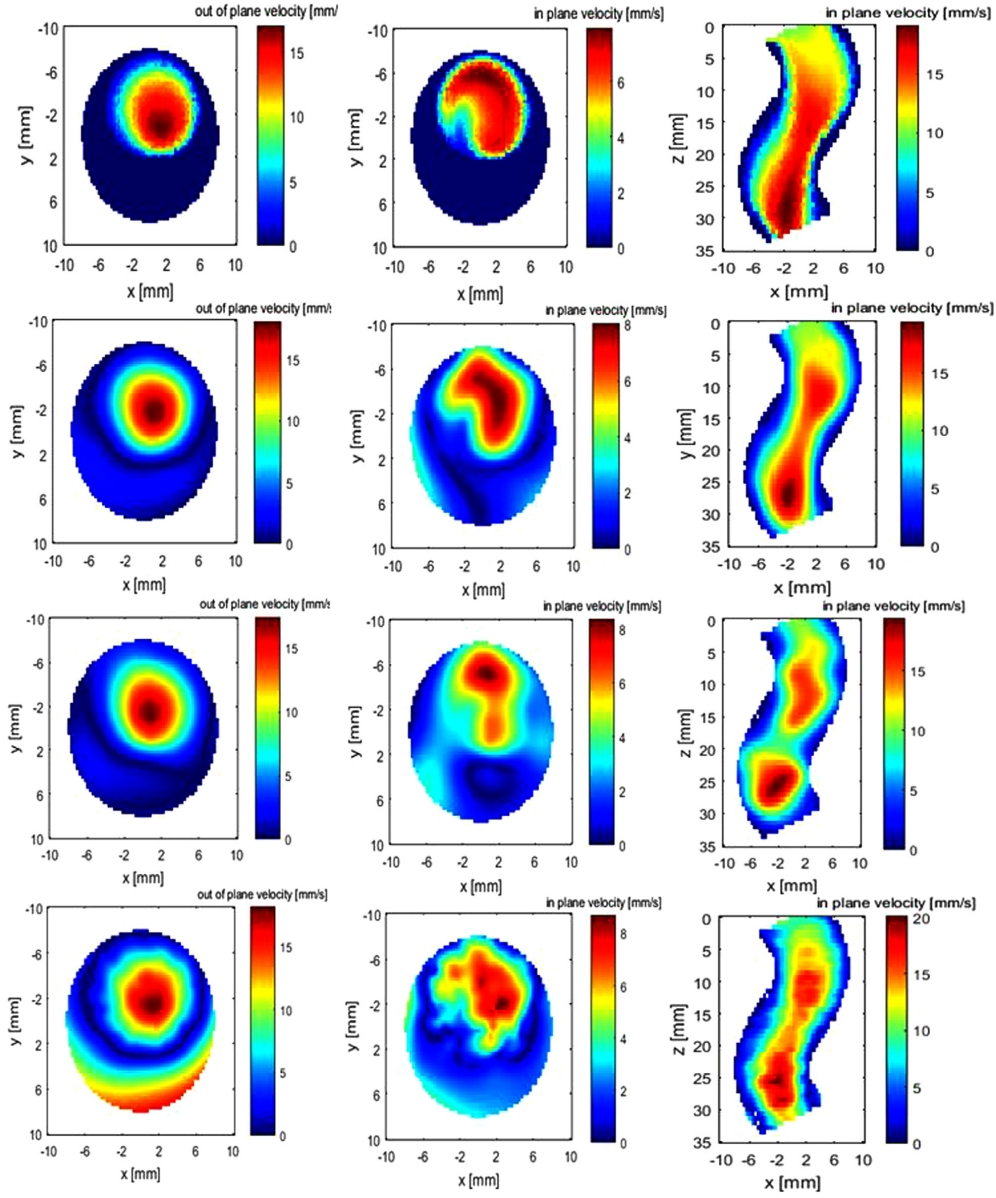
Helical flow. The four rows (from top to bottom) are ground truth by computational fluid dynamics, reconstructed flow field using Gaussian kernel at spacings of 2 and 8 mm and thin-plate spine at spacing of 2 mm. The three columns (from left to right) are the out-of-plane velocity at the cross plane, the in-plane velocity at the cross plane and the in-plane velocity at the sagittal plane.

The reconstruction results are illustrated in Figures 8 and 9. Table 2 lists the errors using four kernels, and the other three RBFs have higher accuracy than TPS, the mean relative error of which is 14% (spacing = 2 mm).

**Table 2:** Absolute error of four kernels for helical flow reconstruction using noise-free input

Kernel	Error (mm/s)
Gaussian	0.9169
IM (inverse multiquadric)	0.9791
Multiquadric	1.0365
TPS (thin plate spline)	1.4036

#### Reconstruction accuracy for different spacing

**Fig. 10:**
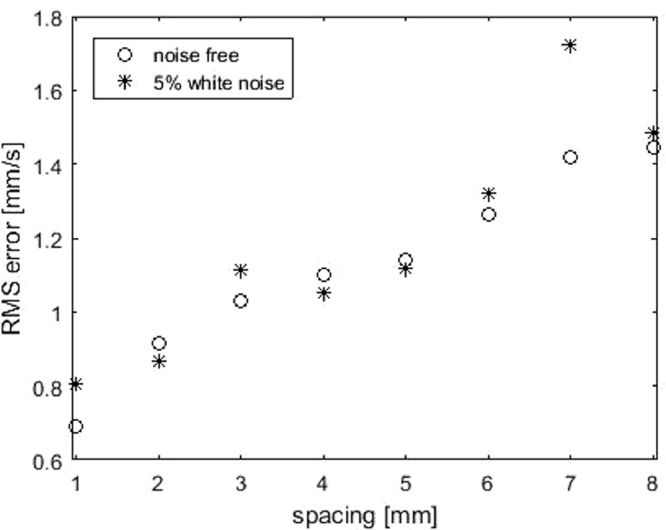
RMS error of helical flow with different spacings using the Gaussian kernel, with no input noise or with 5% white noise. RMS = root mean square.

Similarly to case 1, the error increases with increasing spacing. Figure 10 illustrates the error increases when spacing increases; relative error increases to 14.4% for noise-free input when spacing is 8 mm.

It is interesting to note that reconstruction errors with noise-free input is not always lower than those with noise, which indicates that a sub-optimal regularization parameter is achieved with the L-curve method. This motivates future research exploring other regularization methods.

### *In vitro* experiment case

**Fig. 11:**
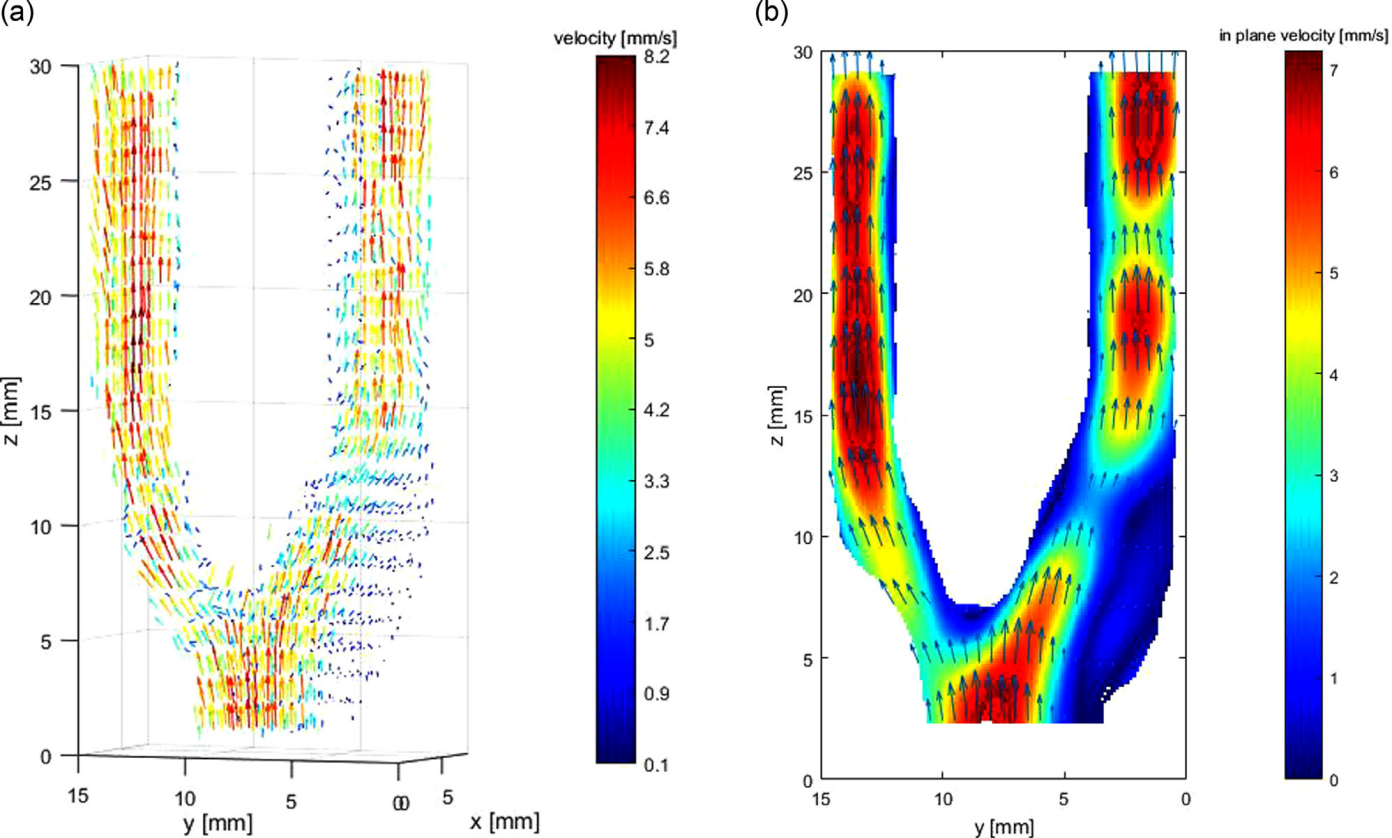
*In vitro* flow experiment: (a) reconstructed steady 3-D flow; (b) velocity magnitude contour at the cutting plane of *x* = 3 mm.

Figure 11 illustrates the reconstructed flow of case 3. We did not conduct CFD because of the difficulty in measuring accurate boundary condition (especially flow inlet condition) and geometry. As a result of the lack of an ideal standard for error evaluation, we did not compare the influence of spacing/kernel on accuracy, and the Gaussian is used for reconstruction. The plane spacing of input is 5 mm.

## Discussion/Summary

This study illustrates the generation of 3-D flow reconstruction through integration of ultrafast plane wave imaging, ultrasound imaging velocimetry, microbubble contrast agents and DFI. It fills a current gap between CFD and experimental ultrasound measurements. By reconstructing a full 3-D flow velocity field with limited samples of 2-D measurements, it can overcome the problem of CFD with real measurement constraints and the problem of current 2-D ultrasound measurements by providing a full 3-D velocity field. Unlike current CFD which usually utilizes only geometry and inlet condition from imaging, UADFI reconstructs the 3-D full velocity field from experimental 2-D in-plane velocity input, can achieve reasonable accuracy and is robust to measurement noise. This method is simple, computationally efficient compared with CFD, mesh free and independent of initial condition and, thus, has potential for faster flow estimation than CFD in biomedical applications. The reconstruction accuracy and reconstruction speed are discussed below.

### Influence of experimental/reconstruction parameters on accuracy

Reconstruction accuracy is related to UIV experimental parameters including spacing, acquisition angles, frequency, compounding angles and UADFI parameters including different RBF kernels, shape parameter (not mentioned in this study) and regularization parameter*.*

It should be noted that the spacing required depends on the complexity of the flow. The proposed method will interpolate the flow field taking into account mass conservation, and recover out-of-plane velocities to generate a full 3-D velocity field, but it would be impossible for the method to retrieve information on a complex local vortex flow if it is not being sampled in the imaging planes. Therefore, we have to sample sufficiently densely (*i.e.,* use smaller spacing) to visualize complex and localized vortex flow, at the cost of slower reconstruction and UIV acquisition. In case 3 of this study we tested the plane spacing at 5 mm for real UIV measurement, and the results were reasonably good. UIV accuracy depends on the acquisition angle, and the higher UIV accuracy is achieved when the angle between flow direction and the acquisition plane is smaller. However, for real acquisition it is difficult to know the flow direction beforehand, and thus, in this study we used two perpendicular acquisition angles to maximize the independence of flow information acquired from two angles. UIV experimental error can be below or around 10% according to Leow et al. (2015), and by optimizing UIV parameters such as the number of plane waves compounded, plane wave tilting angle, frequency, acoustic pressure and pulse length, UIV accuracy can be improved.

Radial basis function kernels also influence accuracy, and a previous study compared Gaussian and TPS kernels with 3-D velocity input (Skrinjar et al. 2009). This study indicated that Gaussian, IM and MU have higher accuracy than TPS. In our study we added 5% Gaussian noise in cases 1 and 2 (17 numerical experiments in total, with different spacing, illustrated in Figs. 7 and 11), and the results indicated the robustness of the method to noise. The shape parameter of the RBF is an open research topic and difficult to optimize explicitly. It is usually found by trial and error or by cross-validation (Fasshauer and Zhang, 2007, Wang and Liu, 2002). In our study we found it by trial and error. The regularization parameter is also vital in increasing accuracy and suppressing the influence of noise, and it can be explicitly optimized with the L-curve method.

### Reconstruction time

For the first two cases, the reconstruction time was <3 min (spacing = 2 mm, number of input vectorial points  < 1000) when data acquisition and UIV post-processing are not included. CFD using Star-CCM+ for case 2 took around 1 h.

The reconstruction time of the third case (spacing = 5 mm, number of input vectorial points  < 3000) was <15 min, including UIV experiment and UIV post-processing. Image acquisition of each plane takes a matter of microseconds, and total US acquisition of the full length takes around 1 min (including movement of stage controller, data transfer and beamforming). UIV post-processing is accelerated by GPU and takes several seconds at each plane. SVD of matrix *G* involves O((3*m*)^3^) flops, and the reconstruction time could be significantly reduced if *m* is reduced, that is, the vectorial data are downsampled or spacing is increased. In addition, an iterative solver with proper regularization has the potential to reduce the reconstruction time to a matter of seconds (not introduced in this study).

### Limitations and future work

One limitation of UADFI is the mechanical translation of the ultrasound probe, as this may be not feasible in some applications, makes acquisition slow and may cause spatial registration error.

Only steady flow is studied. For periodic physiologic flow, one method is to use one probe to acquire flow for a full cardiac cycle ( ≈ 1 s) at each location; then the probe translates to the next location. After acquisition of 2-D flow information of a full cardiac cycle at each location, the velocity field is registered temporally, for example, by aligning the peak 2-D flow rate at different locations temporally or using electrocardiogram gating.

Future work should focus on three areas: shape parameter, Runge phenomenon and better regularization and faster optimization of eqn (9) as SVD is relatively slow. Shape parameter ɛ optimization is not studied here. Although significant research has been conducted to optimize the RBF shape parameter for scalar interpolation (Rippa, 1999, Wang and Liu, 2002), to the best of our knowledge no successful methods for finding ɛ have been reported for DFI so far. According to previous scalar valued RBF studies, when reducing ɛ or increasing the number of data points, the error will first decrease and then increase in finite arithmetical progression (Sarra and Sturgill 2009).

Radial basis functions on a finite interval exhibit wild oscillation near the interpolation boundary, termed the *Runge phenomenon* (Fornberg and Zuev 2007). To evaluate wall shear stress, it is necessary to reduce numerical oscillation near the boundary caused by the Runge phenomenon. Feasible and fast methods to defeat the Runge phenomenon will be further studied, because of the potential to obtain 3-D wall shear stress directly from US imaging.

For regularization, TSVD is relatively slow with large input data but is robust when combined with the L-curve method. To speed up the flow reconstruction process with large-scale data points included in UADFI, an iterative solver should be constructed.

## Figures and Tables

**Fig. 12:**
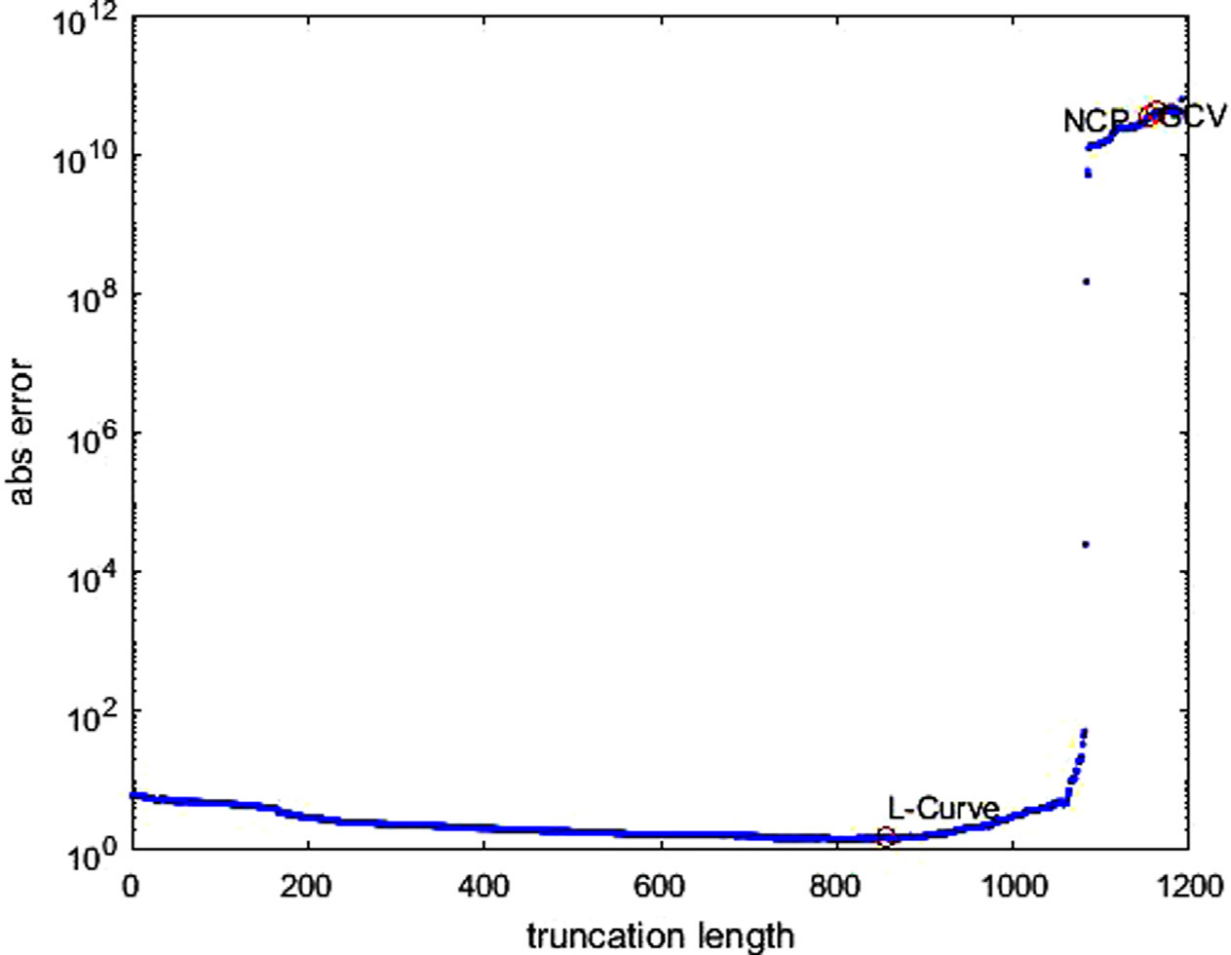
Comparison of L-curve, GCV and NCP using T singular value decomposition in Poiseuille flow case. GCV = generalized cross-validation; NCP = normalized cumulative periodogram.

**Fig. 13:**
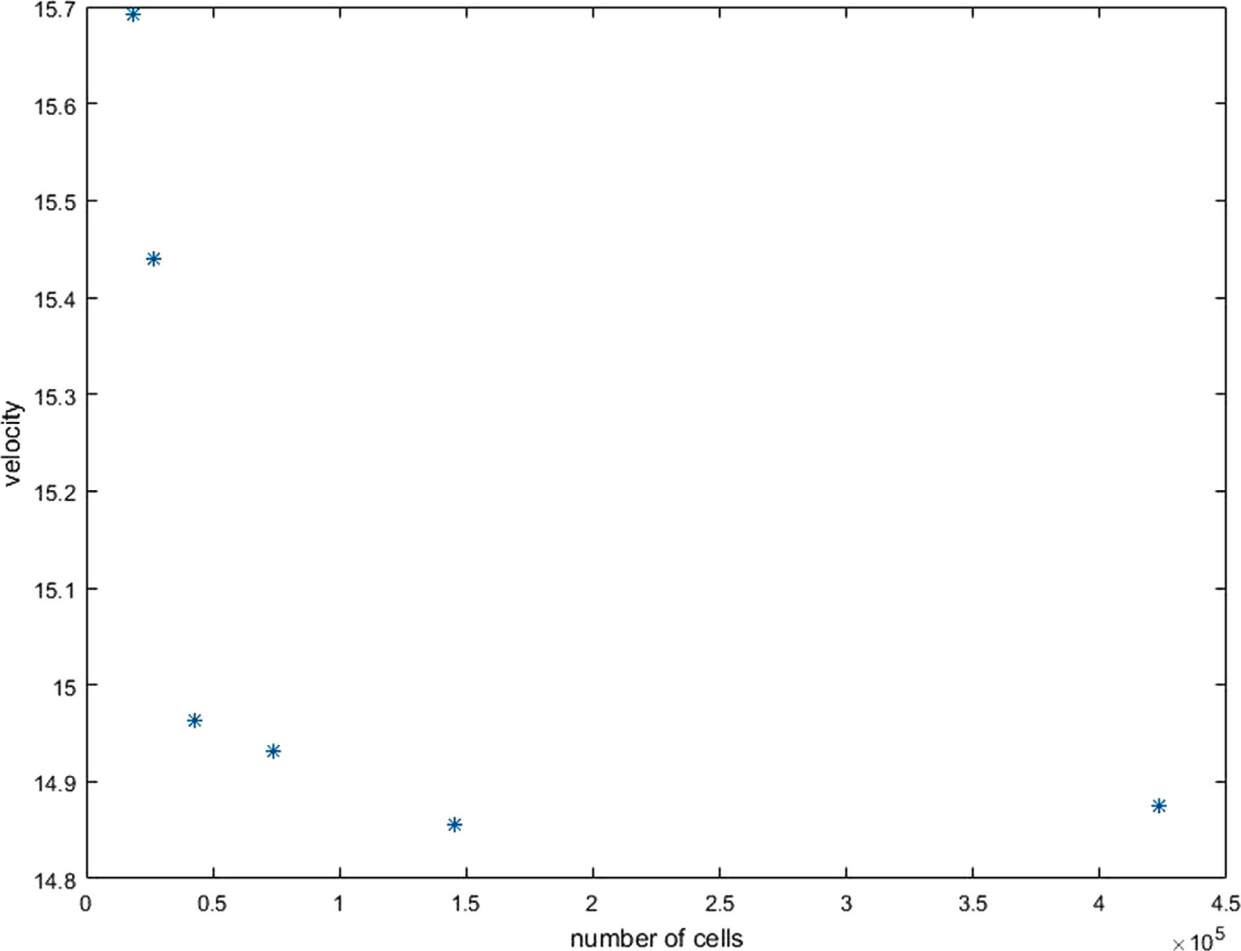
Grid convergence of helical flow computational fluid dynamics.

## Appendix A RBF kernels

Four different RBF kernels used in this study are introduced:

(13) $$\text{Gaussian}:\varphi \left(\vec{r}\right)=\text{exp}\left(-ɛ{\vec{r}}^{2}\right)$$

(14) $$\text{Multiquadric}:\varphi \left(\vec{r}\right)=\sqrt{1+\left(ɛ{\vec{r}}^{2}\right)}$$

(15) $$\text{Inverse}\;\text{multiquadric}:\varphi \left(\vec{r}\right)=\frac{1}{\sqrt{1+\left(ɛ{\vec{r}}^{2}\right)}}$$

(16) $$\text{Thin}-\text{plate}\;\text{spline}:\varphi \left(\vec{r}\right)={\vec{r}}^{2}\text{ln}\left(\vec{r}\right)$$

ɛ in eqns (13)–(16) is the shape parameter and is related to kernel width. The shape parameter has significant influence on the accuracy and numerical stability of the RBF method. In this first study investigating the feasibility of such an algorithm for UIV data, shape parameter optimization was deemed beyond the scope of the current work and was found by trial and error.

## Appendix B Regularization Parameter

Three methods for optimizing the regularization parameter for the TSVD pseudo-inverse method are compared.

A common feature of all regularization methods is their dependence on the regularization parameter to control how much filtering is introduced by the regularization. A proper choice of the regularization parameter is crucial. If prior knowledge of measurement noise level is unknown, three methods are usually used to find the regularization parameter *k*: generalized cross-validation (GCV) (Chung et al. 2008), normalized cumulative periodogram (Hansen et al. 2006) and L-curve (Hansen and O'Leary, 1993, Hansen et al., 2006).

Figure 12 compares the errors of L-curve, NCG and GCV using TSVD with data from case 1. The results indicate that L-curve has highest accuracy compared with NCG and GCV. After random Gaussian noise is added to $\hat{b}$, the optimal regularization parameter is located by the three methods, and the flow fields reconstructed with the three methods are compared with the ground truth. It is of interest to point out that rank(A)=2m (we did not prove it and this study only uses the rank inequality in eqn [8]); when truncation length is >2*m* (1080, see Fig. 12), the error is wildly oscillating because the denominator in eqn (12) is close to 0.

## Appendix C CFD grid independence

Grid convergence was achieved for helical flow by Star-CCM+. Computations were performed on increasingly finer grids until the velocity of an arbitrary point in the centerline of the helix became grid independent, as illustrated in Figure 13.
